# Supplementary material for: Timing and Frequency of Daily Energy Intake in Adults with Prediabetes and Overweight or Obesity and Their Associations with Body Fat
Source: Nutrients. 2020 Nov 13;12(11):3484. doi: 10.3390/nu12113484 (PMC7698054; doi:10.3390/nu12113484)
Supplement: Supplementary file 1 [file nutrients-12-03484-s001.pdf]

Supplementary

Table 1. Characteristics of the participants based on all included participants and acceptable reporters.

	**All participants**			**Acceptable reporters**		
	All (n = 119)	Women (n = 66)	Men (n = 53)	All (n = 81)	Women (n = 45)	Men (n = 36)
Age (years)	57.8 (9.3)	57.9 (8.9)	57.7 (9.7)	58.4 (8.8)	58.2 (8.0)	58.8 (9.7)
Height (m)	1.71 (0.09)	1.65 (0.06)	1.80 (0.07)	1.72 (0.10)	1.65 (0.06)	1.80 (0.07)
Weight (kg)	93.3 (17.8)	86.0 (16.2)	102.3 (15.5)	90.9 (16.5)	84.0 (13.6)	99.52 (15.8)
BMI (kg/m^2)	31.5 (5.0)	31.4 (5.8)	31.6 (3.9)	30.7 (4.0)	30.7 (4.3)	30.7 (3.6)
Body fat (%)	38.0 (7.4)	43.3 (4.5)	31.4 (4.4)	37.3 (7.4)	42.8 (4.2)	30.5 (4.2)
HbA_{1c} (mmol/mol)	41 (2)	41 (2)	41 (2)	41 (2)	41 (2)	41 (2)
Daily energy intake (kJ/day)	8246 (2578)	7402 (2301)	9299 (2523)	9282 (2308)	8356 (2106)	10440 (2017)
Daily eating window (hours)	12.3 (1.8)	12.2 (1.6)	12.3 (2.1)	12.4 (1.5)	12.4 (1.7)	12.49 (1.8)
Number of meals (meals/day)	5.6 (1.5)	5.6 (1.3)	5.7 (1.8)	5.8 (1.4)	5.7 (1.8)	5.9 (1.6)
Time of first intake (hours)	8.23 (1.3)	8.36 (1.1)	8.07 (1.4)	8.15 (1.0)	8.28 (0.9)	8.0 (1.1)
Time of last intake (hours)	20.41 (1.3)	20.46 (1.3)	20.35 (1.2)	20.59 (1.3)	20.67 (1.3)	20.49 (1.3)

BMI, body mass index; Body fat (%), body fat percentage; Data are presented as mean (standard deviation). All participants, all included participants in the study. Acceptable reporters, participants who did not underreport their average daily energy intake.

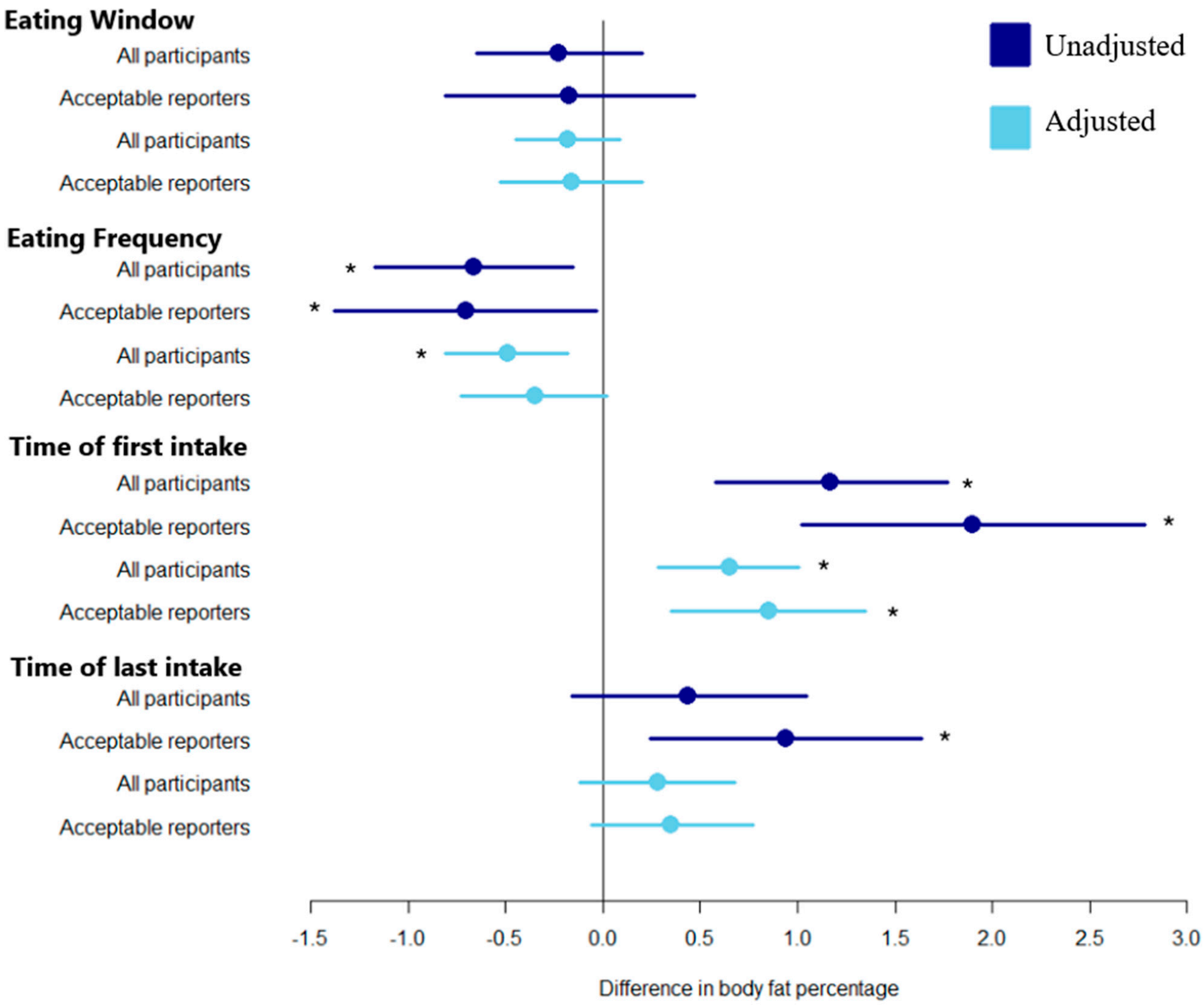

Figure S1. Daily eating patterns and body fat percentage in all participant and in acceptable reporters. Difference in body fat percentage in all participants and in acceptable reporters for each increment in exposures for different daily eating patterns including eating window, meal frequency, time of first intake and time of last intake. An increment in eating window is one hour. An increment in meal frequency is one meal. An increment in time of first intake and time of last intake is one hour. The circles represent effect sizes; extended lines show 95 % confidence intervals. Acceptable reporters, participants who did not underreport their average daily energy intake. * P-value < 0.05. The model is unadjusted and adjusted for age, sex and energy intake.
